# Veterinary Interventions to Improve Neonatal Survival on British Beef and Sheep Farms: A Qualitative Study

**DOI:** 10.3389/fvets.2021.619889

**Published:** 2021-02-04

**Authors:** Katherine E. Adam, Ann Bruce, Alexander Corbishley

**Affiliations:** ^1^Innogen Institute, Science Technology and Innovation Studies, School of Social and Political Science, University of Edinburgh, Edinburgh, United Kingdom; ^2^Royal (Dick) School of Veterinary Sciences and the Roslin Institute, University of Edinburgh, Midlothian, United Kingdom

**Keywords:** health inequalites, veterinary service delivery, neonatal survival, antibiotic usage, qualitative

## Abstract

Neonatal lamb and calf deaths are a major issue in UK agriculture. Consistent mortality rates over several decades, despite scientific advances, indicate that socioeconomic factors must also be understood and addressed for effective veterinary service delivery to improve lamb and calf survival. This qualitative study utilised semi-structured interviews with vets and farmers to explore the on-farm mechanisms and social context, with a particular focus on the role of the vet, to manage and reduce neonatal losses in beef calves and lambs on British farms. Data were analysed using a realist evaluation framework to assess how the mechanisms and context for veterinary service delivery influence survival as the outcome of interest. A lack of a clear outcome definition of neonatal mortality, and the financial, social and emotional impact of losses on both vets and farmers, are barriers to recording of losses and standardisation of acceptable mortality levels at a population level. Despite this, there appears to be an individual threshold on each farm at which losses become perceived as problematic, and veterinary involvement shifts from preventive to reactive mechanisms for service delivery. The veterinarian-farmer relationship is central to efforts to maximise survival, but the social and economic capital available to farmers influences the quality of this relationship. Health inequalities are well-recognised as an issue in human healthcare and the findings indicate that similar inequalities exist in livestock health systems.

## Introduction

Neonatal deaths of lambs and beef calves are considered to be a major problem in UK agriculture. To date, the literature around neonatal losses has focussed mainly on the technical aspects of improving survival. The basic science of losses is well-understood, with management practices on the farm having the greatest impact on survival. A comprehensive review of neonatal survival in small ruminants (1) found that the despite improvements in the scientific evidence base, survival has not increased notably in the last three decades, with mean mortality of around 15%, although this varies widely. The evidence base around mortality levels in a UK-specific context, as well as targeted interventions and management practices that could increase survival, is limited. The most recent study of on-farm risk factors for lamb mortality in the UK was conducted over 20 years ago (2) and no similar risk factor studies have been published for suckler calf mortality.

Translating knowledge around how to increase survival into action and results is an ongoing challenge, indicating that there may be more intangible barriers to meaningful change. There is limited information available in the extant literature on the social, cultural and economic factors related to neonatal survival on beef and sheep farms. A study of Australian sheep farmers found that they had positive attitudes towards improving lamb survival and that social norms and perceived control over survival influenced farmers' behaviour (3). Preliminary qualitative work with UK farmers has identified human factors such as staffing, skill levels and communication as important barriers to reductions in lamb mortality (4). Dwyer et al. (1) found that interventions involving farm staff had a greater impact on increasing survival than the facilities on the farm. The review concluded that facilitative approaches involving farmers and advisors appear to be a promising strategy to reduce neonatal mortality, and that there is a need for application of social science methods to explore this further.

The veterinarian is identified routinely as the main source of information and support on animal health issues (5–7). There is a substantial and increasing body of literature in the social sciences around the role of the farm vet (8). The extant literature can be divided broadly into studies of the role of the veterinary profession in society (9–11), or more contextualised studies of the farm vet's role in addressing specific issues on farms, such as biosecurity (5, 12, 13), antibiotic use (7, 14–16) or infectious disease control (17, 18). Veterinary service delivery is highly contextual, depending on the production system, the disease or management challenge to be addressed, and the individual farm—hence the novelty of this exploration of the role of the farm vet in addressing neonatal losses on beef and sheep farms.

Previous research into veterinary interventions on farms has been conducted in the more intensive livestock sectors. Veterinary involvement in beef and sheep production has received limited attention. In particular, the advisory role of the veterinarian in beef and sheep production does not appear to be well-defined (19). Sheep farmers in the UK regard their veterinarian primarily as a provider of reactive, fire-fighting services (20) and the challenge of engaging farmers in preventive services is well-recognised by vets (11). This can be a source of job dissatisfaction for vets working with farm animals, contributing to farm vets pursuing other career paths and consequent workforce issues in rural veterinary practice (21). Low veterinary involvement on beef and sheep farms has been identified as a key challenge in achieving reductions in antibiotic use, with neonatal loss as one of the major drivers of high antibiotic use (22). The farm business income from grazing livestock enterprises in the UK is significantly lower than all other livestock production sectors (23), which may restrict the resources available to invest in veterinary input. Beef and sheep production is often situated in marginal areas of low livestock density, where it is more difficult to provide a viable farm animal veterinary service (24).

The extant literature commonly identifies that both personal factors and broader social, political and economic influences interact to determine vet and farmer attitudes and actions around animal health and welfare. A realist evaluation framework, based in a scientific realist evaluation approach, was therefore used to analyse the interview data, as “a social theory about individuals being in society—how individual and society are related and the possible interactions between them that might bring about or hinder change” (25). Neonatal survival is both a biological and social phenomenon, and the scientific realist evaluation perspective avoids either an entirely positivist or relativist epistemological position. The goal of realist evaluation is to identify “what works for whom in what circumstances… and why” (26). Veterinary intervention in relation to neonatal survival is viewed here as a social program, or social system, defined as an “interplay of individual and institution, of agency and structure, of micro and macro social processes” (27). Such a system can only be understood by understanding the social rules and institutions that constitute the system. An explanation of a social process from scientific realist evaluation is summarised by Pawson and Tilley (27) as a simple formula: Outcome = Mechanism + Context. The study aimed to understand what vets and farmers do to reduce neonatal survival (mechanisms), why they do it (context) and why it works, or not, to manage and reduce neonatal losses in beef calves and lambs on British farms (outcomes). The analysis also drew briefly on the theory of health inequalities. It has been well-established in human epidemiology that socioeconomic factors influence access to healthcare and health outcomes (28), but not whether such inequalities among farmers influence animal health. The research was conducted as part of a wider project to develop an evidence base to inform practical strategies for enhancing neonatal survival, with the goal of developing a national neonatal survival plan for the UK.

## Methods

Individual face-to-face interviews were selected as the most appropriate method for data collection due to the sensitive nature of the topic of neonatal losses, and to permit more in-depth discussion of the situation with individual farmers and veterinarians. The study was subject to ethical review and approved by the University of Edinburgh School of Social and Political Science Research Ethics Committee (ID: 250518). The interviews were conducted by authors KA and AB. Both are interdisciplinary social science researchers with previous experience of conducting qualitative research with vets and farmers, and are women. KA trained as a veterinarian and AB has a background in livestock genetics and innovation studies. Most of the participants were not known to the researchers prior to the study, with the exception of two vets who were known socially to one of the interviewers. Separate interview schedules were used for the veterinarian and farmer interviews, but covered the same main topics, relating to participants' experiences of neonatal losses on their/their clients' farmers and informed by the realist evaluation framework. Copies of the interview schedules are available from the corresponding author on request. Two pilot interviews were carried out and have been included in the data, as they did not lead to any meaningful changes to the interview schedules.

Veterinarians were recruited through five veterinary practices via contacts of the project lead (author AC). A named veterinarian in each practice was approached via email and invited to take part in the study, and was the point of contact for farmer recruitment. One practice agreed initially to take part, but then later withdrew before any interviews took place. Another practice was approached, but declined to participate. Once the interviews had begun, none of the participants dropped out. The practices that participated are all large, multi-veterinarian practices with a high caseload of beef cattle and sheep work. Three are independently owned, one is linked to a university and one is part of a larger veterinary business group. Two practices are located in Scotland and three in England. The operational area of one of the English practices extends across the border into Wales, and one of the Scottish practices also covers part of the north of England. The veterinary practices were asked to select farm clients to participate, and to include those who had experienced both high and low levels of neonatal losses. This clustered approach to recruiting vets and farmers within practices was used in order to gain access to both perspectives around the veterinary role in neonatal survival.

Semi-structured interviews were conducted with a total of 12 veterinarians (9 women and 3 men) and 16 farmers (4 women and 12 men) across 13 farms. For three of the farmer interviews, two farmers were present, where both interviewees worked together to run the farm. The farm vet was also present for the three farmer interviews within practice A, but did not take an active part in the interviews. Each participant was interviewed only once. Seven of the farms had both suckler cattle and sheep, three had cattle only and three had sheep only. Most of the veterinarians were farm vets and did not provide services to other species, with the exception of two of the Practice D veterinarians, who did some companion animal work. The vets' level of experience ranged from recent graduates who had qualified in the last 2–3 years, to practice principals with several decades of experience in farm practice. There was substantial overlap in the roles of the vets and farmers, with multiple vets also involved in farming in their personal lives, and a number of vets within the participating farmers' families. One of the farmers interviewed had trained as a veterinarian, but was no longer practising. Farmer interviews took place on the farm, usually in the farmhouse kitchen, with the exception of the farmers from practice C, where the interviews took place at the veterinary practice. All veterinarians were interviewed individually at the veterinary practice, in an empty consulting room or break room.

The interviews were audio recorded with the participants' informed consent and field notes were made during the interviews. The audio recordings were professionally transcribed. The mean duration of the interviews was 43 min, with a minimum of 22 min and maximum of 75 min. Data collection ceased and no further interviews were conducted when all of the researchers were satisfied that data saturation had been achieved, on the basis that the interviewers were not hearing new information in the final interviews (29). The transcripts were coded by the two interviewers (KA and AB) and the coding framework was agreed by both. The results are presented under the three main areas of the realist evaluation framework—context, mechanism and outcome—and based on the codes identified under each heading. Coding was carried out using QDA Miner Lite software (V.2.0.5, Provalis Research, Montreal, Quebec, Canada). The COREQ (COnsolidated criteria for REporting Qualitative research) checklist (30) was used as a guide for reporting the study.

## Results and Discussion

An explanation of a social process from scientific realist evaluation is sometimes presented as a simple formula: Outcome = Mechanism + Context (27). The results are therefore presented and discussed in this order, with the outcomes of interest first, in order to frame clearly the challenge of neonatal losses and the goal of maximising survival. The mechanisms of veterinary service delivery and farmer implementation to support these outcomes are then explored, followed by the contextual factors which influence veterinary interventions and ultimately, lamb and calf survival.

### Outcomes

Mortality may appear to be a clearly defined and easily quantified outcome, but the data demonstrated that the level of losses observed on farms were often more of a reflection of the experiences and perceptions of the farmer. As the primary outcome of interest, attempts were made to characterise the vets' and farmers' definitions of the term “neonatal losses.” These definitions were generally framed as cut-off values of the age and stage where a lamb or calf becomes and ceases to be a neonate. The interviewees provided a wide range of definitions, ranging from abortions at any point from scanning onwards through to deaths of lambs and calves up to weaning. However, immediately after birth and the first 48 h of life were generally agreed to be the neonatal period by all participants. For many of the farmers whose sheep or cattle were born indoors, the end of the perceived neonatal period coincided with turnout, when their ability to intervene immediately came to an end. Later losses or diseases such as joint ill were often linked to earlier events in the neonatal period or pregnancy, and some infectious diseases cause both abortion and neonatal death, which further blurred the boundaries. The vets frequently quoted the given definition of a neonate from farm assurance recording (a certification process to demonstrate that production standards are being met on the farm), or from their training, but provided a qualifying statement to the effect that this did not necessarily match their own experience.

The variation in the definition of neonatal losses is likely to be a barrier to efforts to enumerate mortality with a view to setting targets and implementing strategies to improve survival. The data indicated that the level of losses experienced is determined on the basis of the perceived impact, rather than as part of an objective process of counting and recording mortality within clearly agreed parameters. This is explored further within the mechanisms of recording neonatal losses.

#### Impact

The social outcomes of neonatal losses relate to their economic and emotional impact. There is a clear financial cost associated with the death of lambs or calves, resulting from both the loss of production and the costs of investigating and addressing the causes of mortality. In general, calf losses have a greater individual cost due to the higher value of a calf in comparison to a lamb. At a flock level, losses in sheep flocks were felt to result in higher overall losses, as a higher proportion of lambs do not survive. Live lambs and calves are the end product of sheep and beef suckler enterprises, and every loss has a direct impact on the farm's finances. While the financial aspect of losses is clearly important, the emotional impact must not be underestimated. The farmers reported feelings of frustration, guilt, self-blame and depression in response to losses, as well as fear of judgement from other farmers due to the stigma associated with deaths of lambs and calves. The emotional and financial impacts arising from neonatal mortality are closely intertwined, reflecting the dual roles of livestock farmers, as both “*empathetic carers and economic producers of sentient commodities*” (31). The vets experienced the emotional impact of losses when assisting with a lambing or calving which did not result in a live birth, or when dealing with high levels of losses in a clients' flock or herd. They also observed the negative impact of losses on their clients' well-being.

“*I just don't like losing things, and I take it really, really badly when I do, and it's not about money, it's more about you want everything to do as well as it can.”* (Farmer A3

“*It's never nice seeing dead things, especially dead baby animals. It is unpleasant and it feels like it's such a waste.”* (Vet E1)

The cause of losses affects how those involved are impacted. Losses due to circumstances considered to be unavoidable, such as accidents or severe weather, were felt to be more acceptable than deaths which farmers or vets believed that they could have taken action to prevent. The vets and farmers interviewed often recalled the losses that they or their clients had experienced in the previous year, and some farmers were able to provide a detailed description of each individual death, with a final judgement on whether or not the loss could have been avoided. This suggests that reflecting on losses can be a process of continual growth and improvement. Some farmers, however, preferred not to consider their losses too closely, or viewed them as an unavoidable side effect of their production system.

“*If you admit you've got losses you're admitting that you're… almost admitting failure… it's quite hard to do, I think, for some folk. And rather than look upon it as well, I failed… I'm going to learn what I did wrong to make it better.”* (Vet D3)

“*You think you should have done something, or could have done something, or what could you have done to stop that happening.”* (Farmer C3)

Tolerance for neonatal losses varies widely among farmers. Some level of loss is inevitable, and is therefore accepted and normalised, particularly in sheep. As with the definition of neonatal losses, interviewees quoted industry benchmarking figures as the official threshold for “acceptable” mortality, but viewed this with scepticism as not reflecting the individual circumstances of each flock or herd. Farmers with smaller numbers of animals pointed out that even a small number of absolute losses can result in a high proportion of losses. This “trigger threshold” at which losses within a herd or flock are perceived as problematic appears to be highly individual and contextual. Rather than a firm, quantitative proportion or absolute number of animals lost, it was described as more of a qualitative phenomenon, based on the individual farmers' experiences, the circumstances on the farm, the cause of the losses and the perceived impact. The lack of clarity around the definition of “neonatal losses” is also likely to contribute. Veterinarians commented on the variability of the threshold that they had observed in their clients, with some deeply concerned about every individual loss and others apparently unperturbed by considerable mortality. This “trigger threshold” is as a key determinant of the actions taken by farmers and vets in relation to survival, and particularly in the nature of the veterinarian's involvement.

“*We'd like a percentage cut-off, that kind of target as a vet but a farmer doesn't really see that.”* (Vet B2)

“*One of the problems I think that there is that farmers don't talk. You're not going to say to people, by, we had 40 calves dead this year, you're not going to do that… so the threshold of what's normal, it depends on the farm.”* (Vet A1)

“*With lambing as far as I'm concerned, if I stand at 150 per cent and lose 20 per cent it's a disaster, because you can't just have a figure that fits all… that's just someone that's never set foot on a farm quoting figures, at you, isn't it?”* (Farmer A2)

### Mechanisms

Data recording around neonatal losses is an important mechanism contributing to the delivery of veterinary services to reduce mortality. Recording of neonatal calf losses was reported by the participants to be generally good, due to the requirement to record cattle births and deaths with the British Cattle Movement Service. In sheep flocks, record keeping varied among the farmers interviewed from no recording at all to detailed electronic record keeping. Recording of losses is required by farm assurance schemes, although vets expressed doubts about the accuracy of the data recorded. Those who do record mortality tend to keep detailed records about all aspects of their enterprise. Farmers that don't perceive a problem with neonatal losses may not see the need to record, despite the potential value of records to the farm vet.

In order to provide an objective overview of the survival outcomes on farms and to respond effectively, vets need access to records of the losses that have occurred, such as how many lambs or calves were lost, and when and where this happened. While the farmer usually has detailed knowledge of what has happened in their flock or herd, a vet coming on to the farm will not have the same experiential knowledge. Recorded data are therefore especially valuable to the vet to enable them to gain a rapid insight into the issues on the farm. In this way, simple quantitative records of losses can be viewed as an outcome in their own right, as a representation of the situation on the farm, but may not provide the more detailed contextual information that determines whether mortality levels are of concern.

“*I've got a really well run farm, that is all outdoor lambing… and they don*'*t really record anything, but they*'*re really on the ball with everything. But they wouldn*'*t be able to tell you what their neonatal survival rate was.”* (Vet A2)

“*With the sort of things like Red Tractor Assurance and stuff like that… Most of them, they just make it up. They haven*'*t a clue. It*'*s a guess. How valuable that actually is for me and the farmer to share, it*'*s hard to get them really engaged in it…”* (Vet D1)

“*We don't know when an animal's died. We as vets, we have no idea of the mortality on a farm. Yes, you can check on BCMS but can you tell if it's died, sold, slaughtered, you know? You can't. It's just off farm.”* (Vet B2)

Attempts by vets to encourage more farmers to start recording losses have been largely unsuccessful, despite practical efforts to overcome the difficulties of taking notes in cold and dirty conditions while busy with other tasks. Those who were already recording considered it to be a valuable opportunity to learn and improve, but those who were not recording deaths often viewed this as depressing and frustrating. The recording required of farmers, and particularly those with multiple enterprises such as livestock and arable, is extensive. Some degree of “recording fatigue” is perhaps therefore unsurprising. The stigma around losses can prevent effective recording, as farmers fear judgement from peers, including their vet. The negative emotional impact of losses can be substantial, and by recording losses, farm staff are forced to face the associated negative emotions. The most extreme example given across the interviews was from one farmer who had lost a friend to suicide and believed that recording ewe losses had contributed.

“*Sometimes, that*'*s a barrier to people, because if they start recording stuff, they*'*re worried about whether they*'*re actually doing awfully. They would be embarrassed by it… It*'*s sometimes better to bury your head in the sand.”* (Vet A2)

Given the substantial practical and psychological barriers identified to recording data around losses, farmers need to see a clear benefit. Records are used predominantly by farmers to review the previous year's events, with a particular focus on breeding and culling decisions about individual animals. During the interviews, several farmers produced paper records of lamb or calf losses for the previous year and talked through the circumstances of each loss. Some farmers also did this without any prompting from written records and were able to recollect losses that had occurred in detail. As the farmers reviewed their losses, they effectively conducted a “verbal autopsy,” describing the circumstances of each death in detail. The goal of this process appeared to be to assess what caused each loss, and whether the loss could have been avoided. This approach is used as a formal process to determine the cause of death in low resource settings in human healthcare (32) and a similar strategy may be of value in a veterinary context. In contrast to the focus on individual animals by farmers, vets appear to view records of mortality as a resource to identify problems to be addressed at a herd or flock level. This divergence in the purpose of the records may be an additional barrier to recording for farmers, and to effective use of records to determine further interventions. The value of the data to the vet is to provide insight, but they must be able to demonstrate to the farmer how this insight enables them to provide value to the farmer.

“*If you're going to put something down on paper, you'll do it if it's some use to you. If it isn't some use to you, you find something else to do, or something more important rather than log it all.”* (Farmer A1)

#### Veterinary Services

Veterinary involvement in neonatal survival can be broadly divided into the delivery of preventive and reactive services, according to whether the trigger threshold of losses for that farm has been exceeded. Vets and farmers work to prevent losses when mortality is deemed to be within acceptable limits, but when the trigger threshold is crossed, vets and farmers will react to address the problem. As described, this threshold is not easily defined in the absence of context, but has a clear effect on the actions taken. In relation to neonatal losses, the definition of “preventive” and “reactive” veterinary services may differ from a more general context. For example, the veterinarian may attend a sick animal or challenging obstetric event, which would be regarded generally as a reactive service. In relation to survival, the vets' role may in fact be preventive, as their intervention aims to save the life of the dam and/or offspring. For this reason, the vet's role around survival is classified here into preventive and reactive services at the herd or flock level, and individual animal services, such as lambing, calvings, caesareans and attending to sick animals.

##### Preventive

The foundation of neonatal survival is good animal husbandry, delivered consistently by the farm staff. Preventive action is viewed by both vets and farmers as an ongoing process rather than a single event: for example, maternal nutrition during pregnancy affects colostrum production and calf/lamb vigour, which then influences susceptibility to infection and potentially mortality. Lambing and calving is viewed as the peak of the annual production cycle, with the focus on birth and immediate care of new-born animals. Ongoing preventive efforts ensure that considerations such as nutrition, herd/flock health and shelter are managed throughout the year. While this may seem simple, it is not necessarily easy to implement.

“*Nutrition, getting that right, hygiene and colostrum, if you get those three things right, I generally think most of it probably follows itself.”* (Vet B2)

“*Sometimes I think we get away from the basics too far and we've just got to go back to the basics.”* (Farmer B3)

“*I would see people who've have, who've historically had bigger losses and actually most of it is improving management… vaccines and treatments have a place, but most of it is improving management.”* (Vet D3)

Preventive veterinary services are delivered when there are no immediate issues with losses, but ongoing action is supported to maximise future survival. General herd/flock health, such as control of infectious disease in breeding animals, was identified by farmers as an important aspect of survival. Vets' preventive work is therefore centred around advisory services. Health planning is carried out routinely, and as neonatal survival is so intrinsically linked with the general health and management of the herd/flock, is an integral part of herd/flock health planning. An effective health plan must target the most likely issues that will be faced on each farm, which is where detailed veterinary knowledge of the farm enterprise is needed. Raising the issue of mortality can be challenging, due to the stigma and emotional impact of neonatal losses. If the farm has no history of concerning levels of losses, the emphasis can remain on general good practice and any other issues identified. However, if the farm has a history of neonatal losses, or particular risk factors for mortality have been identified, then losses may need to be discussed openly. An excessive focus on negative issues, such as mortality, could damage the constructive working relationship that vets aim to foster. Framing conversations around “survival” rather than “mortality” may help to keep the discussion more solution-focussed.

“*It's better to ask a positive question than a negative but unfortunately as vets, the majority of our work comes off negative. We're trying to change it. We're doing more nutrition, trying to do more promotion, health brands and positives but unfortunately we deal with death*.” (Vet B2)

“*My vet would sit and talk about all the problems we have and then try and come up with a really good plan, but since we started doing that, we seem to have a lot more problems.”* (Farmer A3)

Client education, such as delivering training to farmers and supplying information via practice newsletters, are also important preventive services. All of the practices held regular evening meetings and events for their farm clients as an opportunity for vets to present information and for farmers to share their own experiences. One vet described a recent meeting on neonatal survival that been particularly well-attended and received. Despite the stigma around neonatal deaths and farmers' reluctance to make their losses public, farmers appear to be comfortable discussing the mechanisms for maximising survival, if not the outcomes. Peer-to-peer interactions between farmers are well-recognised by vets as a motivator for change and may be an important source of information for farmers seeking to reduce mortality. Some vets reported encouraging clients to speak to other farmers who have had similar experiences with neonatal losses in order to support their efforts to change practices on farms. The main challenge with this type of event is the limited audience and engaging with farmers who do not attend, although discussions between farmers outside the practice may disseminate the relevant information further.

“*We for example, put on meetings, you know, client education things and it will be the same people that come to all the meetings. But maybe the ten per cent of people that never come to any meetings are potentially, the ten per cent of people that would benefit the most from being there. But it's how you convert those ones, that's the tricky thing, I think.”* (Vet A3)

##### Reactive

If the level of losses is deemed to be unacceptable, action is then taken. Farmers may react independently and make changes to their management systems, or they may involve their vet. This indicates that the tipping threshold between preventive and reactive approaches is not a single cut-off point, but a more incremental approach to addressing losses, starting with the farmer's own actions and escalating to veterinary involvement. The point at which the vet's services are sought depends on each farmer's tolerance of losses, as described previously, as well as other contextual factors. The stigma and fear of judgement around losses appears to extend to the relationship with the vet, and can be a barrier to timely and effective veterinary involvement. One vet was called out by a farmer who was losing a lot of lambs, but found him unwilling to admit to there being a problem when she arrived. Another recent graduate found that farmers were sometimes reluctant to discuss losses, but would do so with a more senior vet where a trusting relationship had been established over a number of years. The vet's approach to discussing neonatal losses must recognise that this is a sensitive topic.

“*You get there and you're like, how many have you lost? ‘Oh, well, we don't want to think about that,' and you're like, but you've lost a lot because you rang me.”* (Vet A1)

“*I'm not aware of any other ones that were particularly losing, but then I probably wasn't the person they were going to speak to about that anyway.”* (Vet D2)

“*I would say it's sometimes hard to really understand what the numbers are because clients are not… they don't like to see it as a problem. So sometimes even with us, even as their, we hope, a trusted source of independent advice for a farmer, that there still seems to be a reluctance to always talk it through.”* (Vet D1)

The vets described the process of investigating losses in the herd or flock and emphasised the importance of visiting the farm in person to understand the situation fully. Observing the farm environment and husbandry practices and discussing the situation with the farmer are essential steps to address problematic losses. A farm visit can also provide an opportunity to review records if available, especially if these are paper copies, which can provide a more complete picture of the farm history to build on the snapshot obtained from a single visit. Diagnostic tests and post-mortem investigations are important tools to identify the likely cause of excess mortality. Regional veterinary laboratories and post-mortem facilities often provide these services and support the farm vet's work by offering an expert overview as well as insight into the current problems and trends within the area. However, the difficulty of reporting negative results from testing or further investigations was described, as farmers often perceived that the action taken had been unnecessary, when in fact it had provided valuable information by ruling out certain causes of mortality.

While action can often be taken quickly to address excess mortality, a sudden increase in losses at lambing or calving can be difficult to resolve immediately. In some cases, mortality can be the result of earlier events, such as nutritional problems or disease during pregnancy. The seasonal nature of beef and lamb production exacerbates this challenge. Both vets and farmers are busy and exhausted in the main lambing and calving period, and may not have the capacity to make major changes. Vets will investigate and do what they can to manage losses in the short term, but then plan a review meeting later in the year to discuss what happened and ensure that the same problem doesn't occur next year. Managing farmer's expectations is vital, as there is the risk that a lack of resolution of the problem in the short term could damage clients' confidence in their vet.

“*If it isn't a straightforward diagnosis and it takes a bit of time, you do feel under pressure to fix it quickly and sort it out… You just want to make it stop as quickly as possible and that can be hard, especially because we're at a busy time as well.”* (Vet C2)

Vets are an important source of emotional and mental health support to their farm clients. The emotional impact of neonatal losses on farmers has been described, and the stresses and pressures of the lambing and calving season on both farmers and vets can be intense. Recent, as yet unpublished, research has found that farmers are most likely to seek support for stress and mental health issues from their vet (33). Vets may require additional training and resources to deal with these issues in their clients and protect their own well-being. Both professions have high levels of mental health issues and suicide (34, 35), and a strong, emotionally supportive relationship could have wider benefits for both groups.

“*There are a few farmers that in the last 12 months I've had to sort of signpost to a few help organisations. Yes, it's quite…I don't think it's talked about enough*.” (Vet C1)

“*I went to do a [caesarean] for someone last year and she was coughing up a lung, and I was like, ‘You need to go to the doctors', she said, ‘I haven't got time'. You know, you just feel so sorry for them.”* (Vet D2)

“*We go on farms and sometimes people just want a chat because you might be the first one they've really seen in a couple of days.”* (Vet B2)

##### Individual animal

The vet attending a lambing or calving is the classic image of the farm veterinarian, and is indeed the foundation of the vet's relationship with many of the farms in the study. When farmers were asked about the role that their vet plays in survival, responding to obstetric emergencies was generally the first intervention that came to mind. This was often described initially as the farm's only interaction with the vet, until further probing elicited additional veterinary involvement at a herd or flock level. The question of when to call the vet to a lambing or calving was raised by both vets and farmers, with vets describing situations where they may have had a better outcome if they had been involved earlier. The farmers faced a judgement call between involving the vet when needed and avoiding the additional cost of unnecessary veterinary intervention. They stressed the importance of experience and observational skills when making this decision, as is the case when deciding whether to provide obstetric assistance themselves. The avoidance of guilt and self-blame often motivated farmers to call the vet, as farmers wanted to feel that they had done everything possible. The value of the individual animal will influence their decision, with a greater willingness to involve the vet early for a calving than a lambing, and similarly for a pedigree animal.

“*But that's why, as I say, I get a vet, because if I do it, I'm always wondering if I've made a mistake, if we calve it and we lose it. If the vet calves it and loses it, well it wasn't my mistake, I'm not saying it's the right…but you know what I mean? I know I've done everything I can.”* (Farmer D1)

“*There's no prizes for ringing up… the local knackers.”* (Farmer B2)

These emergency interactions aim to prevent losses at birth, but are also central to developing and maintaining the trusting relationship that permits deeper communication and involvement of the vet in the farm enterprise, with potentially greater impact on survival. For more experienced vets, an emergency call can allow them to gain access to a farm to observe and address any other issues. For more recently qualified vets, attending individual cases is an opportunity for them to demonstrate their competence and get to know the farmer and their farm, with a view to providing herd or flock level services themselves in future.

“*I got called to an animal with choke, a heifer with a choke on Saturday morning, a client we don't go to very often. I don't know them that well, know them well enough, but don't know them that well. Okay, I sorted the choke out, yeah, relatively easily, but that was a great opportunity to go and have a look at his cows, which we hadn't seen for ages. And then we got chatting about, he says, oh well yeah, the cows are really fat and they're coming in and I'm doing this and… a really good conversation, but it was only because I went and had a nosy and got engaged with him that he actually started talking about it.”* [Vet D1]

“*They're very much they're an [Emma] client, they're a [John] client, and so I'll go out and do their emergency work, but generally there is a someone that they would message directly about something in particular, or the person that does their health plan.”* [Vet D2]

##### Antibiotic Use

Antibiotic use as a mechanism for addressing neonatal morbidity and mortality was explored in particular detail during the interviews due to widespread concern about antibiotic use in farm animals and the potential for antibiotic resistance to develop. Beef and sheep producers have some of the lowest levels of use among the UK livestock sectors (36), but are still subject to pressure to minimise use where possible. Preventive antibiotic use was uncommon in neonatal calves in the experience of the participants, but neonatal loss has been identified as “hotspot” for antibiotic use in sheep (22). Oral antibiotics are used widely to prevent and treat bacterial gastrointestinal infections (“watery mouth”) in lambs, and were the main subject of the discussions.

As described earlier, the emotional impact of losses that are perceived as avoidable is higher than those seen as inevitable, and farmers understandably wish to avoid losses that could have been prevented. There is also a financial consideration, as these antibiotics are relatively low cost, particularly when compared to the potential loss of a lamb, or to more systematic changes on the farm. Antibiotics are used in some flocks as a mitigation strategy when other aspects of husbandry are not ideal, and in some situations, are necessary to prevent widespread losses.

“*We usually try and wait until we get a case or two before we do it, but as soon as I get a couple of cases, I'm kicking myself. I hate losing a fit, healthy animal, for, what I think, is something that's preventable.”* (Farmer D1)

“*In some situations where hygiene's atrocious, and colostrum quality is poor, and feeding's not right, then it probably does have a place…”* (Vet A2)

“*Something like Spectam, I think it works out at 17p a lamb, if they don't do that, it could cost them potentially £70 a lamb if the lambs die, so it was a massive risk to them.”* (Vet C2)

Oral antibiotic use in lambs appears to create a sense of reassurance for farmers. The act of giving each newborn lamb an oral dose has been perceived historically as best practice, and as doing the best thing for the lambs to prevent disease. Some vets had introduced strategies that recognised these behavioural barriers to ending preventive oral antibiotic use: for example, one vet had started providing farmers with probiotic paste to be given orally in place of antibiotics, and used a smoking cessation analogy to describe this process.

“*I think it's a lot like smokers, you've got to replace their fixation with something, you've got to give them something else to put in their hand. So quite often probiotic pastes to make them feel like they're doing something.”* (Vet B1)

The drive for reduction in antibiotic use among the interviewees came primarily from the vets, although the farmers are also aware of the pressure to reduce use. There is however a risk that disease and losses can increase when preventive antibiotics are withdrawn, as some of the farmers had experienced, although others have stopped prophylactic use with no ill effects. Vets are well aware of the risks of antibiotic resistance and are working to reduce unnecessary use in neonatal lambs, with some having experienced clinical resistance in their own and their clients' flocks. However, this places the vet in a gatekeeper role, which may damage the supportive relationship that they attempt to build and maintain with each client, particularly if withdrawing oral antibiotic use results increased mortality. Openness and communication through the process is essential.

Working within the classification presented here of the mechanisms for delivery of veterinary services, oral antibiotic use in lambs encompasses both preventive and reactive services. Vets are now working with their clients to encourage a shift away from preventive antibiotic treatment, but recognise that antibiotics can be an appropriate part of a reactive strategy. Antibiotic use presents a paradox, in that vets generally try to encourage their clients to shift from a reactive to a preventive approach. With increasing restrictions on preventive antibiotic use, the opposite is true, with vets pushing for a reduction in prophylactic use and for antibiotics to be used reactively in response to observed problems. This may present an additional cultural barrier to strategies to reduce antibiotic use in sheep production.

### Context

As the mechanisms by which vets deliver services around survival are explored, it becomes clear that the context is highly influential, both on these mechanisms and the ultimate outcome, as outlined by the realist evaluation framework formula of Outcome = Mechanisms + Context. “Context” refers here to the factors influencing what vets and farmers do to reduce losses; essentially, the drivers and barriers to taking action, encompassing the individuals involved, the farm, the veterinary practice, and the social, political and economic environment. As described in the section Outcomes, neonatal losses have both financial and emotional impacts on farmers and their vets. Conversely, the contextual factors which influence the level of losses that occur also encompass economic, social and cultural considerations. It is clear that a trusting vet-farmer relationship is central to effective veterinary intervention, particularly for preventive services at a herd or flock level. The individual relationship with each client is influenced by the circumstances of the vet and the farmer. Understanding the social and economic context in which vets and farmers operate provides valuable insight into this complex relationship, and supports understanding of the mechanisms for veterinary involvement to reduce mortality.

“*I'd say all the vets are really good to be fair. I get on with them all. I probably use [vet's name] more because I find… she knows what's going on on the farm… It's the same with humans. If you go to the doctor's and see one doctor, then the next doctor and you've got to explain it all over again. Whereas if you're using the same person all the time, they know the history of the farm, they know what's going on and everything like that.”* (Farmer C1)

The blurred boundaries between vets and farmers in many rural communities is likely to have a substantial influence on their working relationships. It has been shown that vets from a farming background are more likely to enter (37) and remain in (38) farm practice, but the dual role of the “farming vet” had not been explored in detail. Within the small number of study participants, there was significant overlap, with vets living and working on the family farm, and farmers with a vet in the family. This may be beneficial, providing the vet with an insider's perspective and supporting the trust between vet and client. However, when the vet is also a farmer, they may be viewed less as an impartial advisor and more as a potentially judgemental peer when dealing with sensitive topics such as mortality. The stigma around animal disease and death may inhibit a frank discussion of the farmer's problems.

“*The vet who was originally at [Practice D], his wife was godmother to my youngest, so he was great, and when my husband died 20 years ago, he kind of came in once a week to say, you should be doing this, you should be doing that.”* (Farmer D1)

“*They don't like to look negative in front of their peers and… vets are starting to become peers to farmers.”* (Vet B2)

The role that the individual vet plays in reducing losses on their clients' farms is influenced by their personal experience and characteristics, as well as the context of the practice in which they work. A lack of confidence among less experienced vets is a barrier to the delivery of effective herd or flock level services. The theoretical knowledge required must be combined with an intimate knowledge of the farm enterprise and good communication skills, which take time to develop. Preventive services in particular require different skills to clinical approaches to individual animals, and the stakes are often higher than when dealing with individual animals.

“*It's not my livelihood. I'm still going to get paid. It's their success and failure as a year on my advice… if I'm wrong, it's not my farm at stake.”* (Vet C1)

The client base within the veterinary practice affects the mechanisms for service delivery. Several of the vets interviewed had worked in other practices in different parts of the UK, and described the differences that they had experienced. For example, one vet described working previously in a practice with mainly small family farms, where lambing ewes would be brought to the practice, but that in her current job, with predominantly larger commercial flocks, she would travel to the farms as they couldn't spare a member of staff. The culture within the practice also appears to influence the level and type of veterinary involvement. There were noticeable differences between practices in the way that the vets described their clients and the vet-client relationships. All of the vets interviewed within one practice spoke negatively of farmers who were perceived to be doing a poor job, or who were not willing to engage with veterinary services. However, the vets within another practice emphasised the importance of a supportive, non-judgemental attitude and of working collaboratively with clients.

“*The culture that we have at this practice is a very healthy one in the sense of we are like working together, it's not we're dictating to them and they're not listening or they're saying they know better than us, it is a two-way street which I think is how it ought to be.”* (Vet A1)

Veterinarians are highly motivated to improve survival on their clients' farms, and are keen to increase their involvement, particularly to help farmers to prevent losses before they occur. Working with clients who were unwilling to take the advice given or make changes was therefore a source of frustration. Vets spoke of their clients as being more or less “proactive” on the basis of their willingness to engage with veterinary advice and implement recommendations around survival. Farmers' attitudes towards the health and welfare of their stock also influenced how proactive they were perceived to be.

“*You get frustrated when you start out with people who don't want to listen*.” (Vet C2)

“*You get the odd backwards farmer that is, you know, hanging by a thread, just doing everything because that's the way they've always known how to do it, and they will not be changed. But no, on the whole we do have quite a proactive client base*.” (Vet B3)

At a farm level, the resources available, such as finances, facilities and staffing, affect the actions that can be taken by the farmer to avoid losses and will determine the level of veterinary involvement. The financial resources depend on a number of external factors, such as the market for beef and lamb, and agricultural subsidies, as well as the business model on each farm. Veterinary services relating to neonatal survival are provided privately in the UK, and each engagement with the veterinarian is an economic decision for the farmer. This adds another layer of complexity to the relationship—while effective veterinary interventions, particularly preventive services, are based on trust and understanding, they are also a financial transaction, and veterinary involvement adds costs to production.

The vets often described using economic arguments to encourage farmers to engage with their services, by demonstrating the reduction in disease, lost production or mortality that will result. In some cases, the examples given by the veterinary interviewees demonstrated an economically neutral outcome; the cost of veterinary intervention will equal the loss avoided, but does not necessarily provide a net benefit in financial terms. The onus is on vets to show that their work will add value, not just cover costs. However, the value provided may be more intangible, such as avoidance of the emotional impact of losses or improvement in the farmer's job satisfaction or quality of life. As the emotional and financial value of livestock to farmers are so closely interlinked, decisions about veterinary involvement are unlikely to be purely rational, economic choices.

“*In the greater scheme of things, we don't need to save very many lambs to completely pay for ourselves.”* (Vet D3)

“*if you said, well… you've had 10 lambs that have been stillborn, so that's 800 quid. Well, for that 800 quid we could have blood sampled six of the ewes before the tup went in or lamb scanning. We could've diagnosed anything like enzootic abortion or toxo or anything like that and then, you know, yes, it would've cost you X amount in vaccine but, you know, it would cost you this much, save you this many lambs and it will work out that your profit margin would go up by this much.”* (Vet B1)

“*If we can make survival better then they're going to be in the game for longer, and that will make a positive difference for everybody, as us as well as them. Because we've got clients to service, that need our services, and they're still in business”*. (Vet D1)

Socioeconomic inequalities among beef and sheep farmers appear to be an important factor driving the heterogeneity in engagement with veterinary services to prevent and respond to excess neonatal mortality. Health inequalities are well-recognised in human health as differences in health outcomes due to socio-economic factors (39). The results of the study indicate that similar health equalities may be present in farmed animals, based on the resources available to their keepers. Farms with limited resources may experience poorer outcomes due to less suitable facilities or less scope for investment in health interventions. Alternatively, farmers under greater economic pressure may be more driven to maximise survival and productivity in order to sustain their business. Animal health inequalities may also exist between livestock production sectors in terms of access to veterinary services. The seasonal nature of lambing and calving creates pressure on veterinary resources, resulting in lower availability of veterinary services and farmers' capacity to implement management changes at the most crucial point in the production cycle.

Health inequalities are not only driven by access to financial resources, but by social and cultural capital (40), described in an agricultural context as “*the fundamental principle… that economic and social transactions are promoted through the quality of the interactions within a community or network*” (41) and identified as a crucial component of the vet-farmer relationship on dairy farms (13). The data from the interviews suggest that the “proactiveness” described by vets reflects extrinsic factors, such as the economic status of the farm or the social standing of the farmer in the local community, as well as the vet's perception of intrinsic factors, such as the farmer's personality or motivations. The mechanisms identified for veterinary input to minimise losses relies heavily on connections with vets and other farmers, indicating that farmers' relationships and social standing in their community will impact their access to livestock health services and information. The socioeconomic inequalities identified appear to produce a vicious circle limiting effective service delivery. Farmers with lower levels of social connection or economic capital are least likely to be able to access or implement veterinary advice around survival, which may lead to their being deemed “less proactive” and further reducing their access to veterinary support.

A similar feedback effect can be observed around farmers' mental and physical health, and neonatal losses. The Outcomes section describes how the impact of losses and the pressures of lambing and calving time affect farmers' health and well-being, and several farmers described injuries or illnesses that had limited their ability to care for their animals. A study of health inequalities in Scotland found that they were most pronounced in remote rural areas (42), which will affect many farmers with a subsequent impact on the animals under their care. Strategies to increase neonatal survival must support farmers to protect their own health and well-being as well as that of their animals.

### Study Limitations

Despite efforts to include participants from a range of geographical locations within Great Britain, it must be noted that the participants are mainly located in rural, lowland areas, with limited representation of upland farming systems in remote regions, or peri-urban agriculture close to major centres of population. The participants were not recruited with the goal of obtaining a statistically representative sample from the population, as this would not be appropriate for a qualitative study of this nature. Instead, the participants were selected to include as wide a range of ages, genders, locations and veterinary and farming business models as possible. This type of study requires vets and farmers to be willing to take part in research by providing their time and discussing a potentially sensitive topic. The results are therefore likely to represent a particular typology of vets and farmers who see the value of engaging in research, and their level of knowledge and interest in the topic may be higher than the typical vet or farmer. The presence of the vet during the farmer interviews with Practice A may have inhibited farmers' willingness to talk frankly about their relationship with their vet, but may also have encouraged greater general comfort and openness due to the presence of a trusted advisor. This was taken into consideration when analysing the data.

## Conclusions

Identifying what works, as the central goal of scientific realist evaluation, relies on a clearly defined outcome. This is complicated in the case of neonatal survival by the lack of clarity around the definition of both survival itself and what constitutes success for vets and farmers, as well as the complexity of the social and cultural barriers to effective data recording. The insights gained from the interviews show the limitations of a purely quantitative, target-driven approach. It appears that there is a need to separate population and individual definitions of what works in order to make progress. In terms of achieving reductions in mortality on farm, the “facilitative approach” proposed by Dwyer et al. (1) is supported by the study findings. The vet and farmer must work together to define what success looks like for the herd or flock, before deciding on the mechanisms by which this can be achieved. Research into veterinary service delivery on farms is often framed around the distinction between preventive and reactive services. When this distinction is applied to veterinary interventions around neonatal losses, it becomes clear that these definitions are in fact contextual and dependent on the outcome of interest. The “trigger threshold” on each farm at which losses become problematic and the actions taken by the farmer and vet shift from preventing to reacting, is highly individual. At a population level, current mechanisms for standardised recording of neonatal survival are unlikely to be effective due to the lack of a standardised definition and the emotional and social barriers around recording losses. Innovative approaches to data collection are needed, and novel methods for effective surveillance and benchmarking of survival must demonstrate a clear benefit in relation to the individual farmer's goals for the beef or sheep enterprise.

It has been observed previously that many vets come from a farming background (13, 38), but this study appears to be the first to identify that farm vets are often active farmers in their own right, and that this dual role is likely to affect the services that they provide. These “farming vets” may bring deeper knowledge and understanding to their role as advisors, but also lack professional impartiality, resulting in clients withholding information for fear of judgement. The blurred boundaries between these roles must be considered when formulating intervention strategies which rely on effective relationships and communication between vets and farmers for implementation. There is increasing awareness and interest in innovative communication strategies among farm vets, with collaborative, solution-focussed approaches such as Motivational Interviewing gaining traction (43). Enabling vets to adopt more of a reflexive approach, and to consider their own position, beliefs and practices as clinicians and advisors, and in many cases, also as farmers and members of the local agricultural community, is likely to improve service provision, and in turn, increase neonatal survival.

In order to increase neonatal survival effectively, vets require an evidence-based toolkit of proven, effective interventions—both preventive and reactive—that can be adapted easily to provide individualised approaches, cognisant of the context and challenges on each individual farm. Approaching neonatal survival as a social as well as a biological issue is likely to result in better outcomes.

## Data Availability Statement

The datasets generated in this article are not readily available because the interview transcripts are confidential in order to protect participant anonymity. Requests to access the datasets should be directed to k.adam@ed.ac.uk.

## Ethics Statement

The study involved human participants and was reviewed and approved by the School of Social and Political Science Research Ethics Committee, University of Edinburgh. The participants provided their written, informed consent to participate in this study.

## Author Contributions

KA is the lead author of the manuscript. AB and AC revised the manuscript critically for intellectual content. KA and AB designed the study, collected and analysed the interview data. AC contributed significantly to the design of the work and the acquisition of data. All authors have given final approval to the work to be published and agree to be accountable for all aspects of the work and have made substantial contributions to the conception and design of the research, data collection and analysis, and drafting and/or revisions of the manuscript. As corresponding author, KA takes primary responsibility for communication during manuscript submission, review and publication.

## Conflict of Interest

The authors declare that the research was conducted in the absence of any commercial or financial relationships that could be construed as a potential conflict of interest.
