# Supplementary material for: Veterinary Interventions to Improve Neonatal Survival on British Beef and Sheep Farms: A Qualitative Study
Source: Front Vet Sci. 2021 Feb 4;8:619889. doi: 10.3389/fvets.2021.619889 (PMC7890239; doi:10.3389/fvets.2021.619889)
Supplement: Supplementary file 1 [file Data_Sheet_1.PDF]

Farmer Interview Schedule

AHDB Project: Development of an integrated neonatal survival and sustainable antibiotic plan

**Introduction**	
Tell me a bit about the farm	Particularly your beef suckler herd/sheep flock e.g. breed, numbers, pedigree/commercial.<br>What sort of lambing/calving system do you have? What lambing/calving pattern? Indoor or outdoor? Staff?
How would you define "neonatal" losses?	Up to what age? Would you include stillbirths?<br>Why?
Talk me through a typical neonatal loss in your herd/flock	(How does this differ between cattle and sheep?)
**Current practices**	
What do you do to avoid neonatal losses in your herd/flock?	Before, during and after birth<br>Housing and/or environment<br>Preventive vs. reactive – what triggers action?<br>Information sources?
How does your vet help you to minimise neonatal losses?	Vaccination; medication (antibiotics); herd health/advisory role; post mortems
Do you record data on neonatal losses?	Why/why not? How (paper, electronic)?<br>Any analysis (by you or your vet)?<br>What do you do as a result? Does it make a difference?
**Drivers and barriers**	
How much of a problem are neonatal losses in your herd/flock?	Has this changed over time?<br>What problems does it cause for the business? For you?<br>How do you feel when you lose a calf/lamb?
What motivates you to reduce neonatal losses?	Benefits of reducing neonatal losses? For your business? For you?<br>How much influence do you feel that you have on neonatal survival in your herd/flock? How much is it down to luck?
What are the main challenges of reducing neonatal losses in your herd/flock?	Are there other actions that you would like to take to increase neonatal survival in your herd/flock, but don't at present?<br>What are the barriers to taking these additional actions?<br>What would help you to overcome these challenges?
**Conclusions**	
What do you think would make the biggest difference to improving neonatal survival?	In your herd/flock?<br>In the UK beef/sheep industry as a whole?
What additional research and/or technology is needed to reduce neonatal losses?	Do you have any personal experience of these innovations?<br>What difference would this make to neonatal survival?

Vet Interview Schedule

AHDB Project: Development of an integrated neonatal survival and sustainable antibiotic plan

**Introduction**	
Tell me about your current role in the practice.	How much beef/sheep work do you do?<br>What proportion of your clients have beef cattle, sheep, or both?
How would you define “neonatal” losses?	Up to what age? Would you include stillbirths?
How much of a problem are neonatal losses in your clients’ herds/flocks?	Has this changed over time? How does this vary between herds/flocks? Is it different for beef herds and sheep flocks?<br>What sort of problems does it cause for their business? For them? For you?
**Current practices**	
What do you do to help your clients avoid neonatal losses in their herd/flock?	Reactive “fire-fighting” role – addressing problems<br>Preventative/advisory role: Before, during and after birth.<br>Vaccination.<br>How are antibiotics used to minimise losses (vet/client)?<br>Where do you get information about reducing neonatal losses?<br>How do you share this information with your clients?
Do your clients record data on neonatal losses in their herd/flock?	Why do you think they do this (or not)?<br>How do they record losses (e.g. paper, electronic)? Do you do any data recording? Do you use a post mortem service?<br>What do you do with these data? Do you do any analysis? Does your client?<br>What do you do as a result? Does it make a difference to neonatal losses?
**Drivers and barriers**	
What motivates you to help clients to reduce neonatal losses in their herds/flocks?	How does it affect you personally when losses occur?<br>What are the benefits of reducing neonatal losses:<br>- for your clients? For you?<br>What do you think motivates your clients to reduce neonatal losses?
How much influence do you feel that you have on neonatal survival in your clients’ herds/flocks?	What strategies do you use to change their behaviour?<br>How much is it down to luck?
What are the main challenges in reducing neonatal losses in your clients’ herds/flocks?	Are there other actions that you would like your clients to take to increase neonatal survival in their herd/flock, but don’t at present?<br>What are the barriers to taking these additional actions?<br>What would help you to overcome these challenges?
**Conclusions**	
What do you think would make the biggest difference to improving neonatal survival?	In your clients’ herds/flocks?<br>In the UK cattle/sheep industry as a whole?
What additional research and/or technology is needed to reduce neonatal losses?	Do you have any personal experience of these innovations?<br>What difference would they make to neonatal survival?
